# SNP-Based Linkage Mapping for Validation of QTLs for Resistance to Ascochyta Blight in Lentil

**DOI:** 10.3389/fpls.2016.01604

**Published:** 2016-11-02

**Authors:** Shimna Sudheesh, Matthew S. Rodda, Jenny Davidson, Muhammad Javid, Amber Stephens, Anthony T. Slater, Noel O. I. Cogan, John W. Forster, Sukhjiwan Kaur

**Affiliations:** ^1^Biosciences Research, Agriculture Victoria, AgriBio, La Trobe UniversityBundoora, VIC, Australia; ^2^Biosciences Research, Agriculture VictoriaHorsham, VIC, Australia; ^3^South Australia Research and Development Institute, Plant Research CentreUrrbrae, SA, Australia; ^4^School of Applied Systems Biology, La Trobe UniversityBundoora, VIC, Australia

**Keywords:** legume, pulse, single nucleotide polymorphism, fungal disease resistance, molecular breeding

## Abstract

Lentil (*Lens culinaris* Medik.) is a self-pollinating, diploid, annual, cool-season, food legume crop that is cultivated throughout the world. Ascochyta blight (AB), caused by *Ascochyta lentis* Vassilievsky, is an economically important and widespread disease of lentil. Development of cultivars with high levels of durable resistance provides an environmentally acceptable and economically feasible method for AB control. A detailed understanding of the genetic basis of AB resistance is hence highly desirable, in order to obtain insight into the number and influence of resistance genes. Genetic linkage maps based on single nucleotide polymorphisms (SNP) and simple sequence repeat (SSR) markers have been developed from three recombinant inbred line (RIL) populations. The IH × NF map contained 460 loci across 1461.6 cM, while the IH × DIG map contained 329 loci across 1302.5 cM and the third map, NF × DIG contained 330 loci across 1914.1 cM. Data from these maps were combined with a map from a previously published study through use of bridging markers to generate a consensus linkage map containing 689 loci distributed across seven linkage groups (LGs), with a cumulative length of 2429.61 cM at an average density of one marker per 3.5 cM. Trait dissection of AB resistance was performed for the RIL populations, identifying totals of two and three quantitative trait loci (QTLs) explaining 52 and 69% of phenotypic variation for resistance to infection in the IH × DIG and IH × NF populations, respectively. Presence of common markers in the vicinity of the AB_IH1- and AB_IH2.1/AB_IH2.2-containing regions on both maps supports the inference that a common genomic region is responsible for conferring resistance and is associated with the resistant parent, Indianhead. The third QTL was derived from Northfield. Evaluation of markers associated with AB resistance across a diverse lentil germplasm panel revealed that the identity of alleles associated with AB_IH1 predicted the phenotypic responses with high levels of accuracy (~86%), and therefore have the potential to be widely adopted in lentil breeding programs. The availability of RIL-based maps, a consensus map, and validated markers linked to AB resistance provide important resources for lentil improvement.

## Introduction

Lentil (*Lens culinaris* Medik.) is a self-pollinating, cool-season, grain legume crop that is produced throughout the world and is valued due to its high protein content. However, lentil production is limited by a number of abiotic and biotic stress factors (Erskine et al., 1994), and fungal diseases of particular significance are ascochyta blight (AB), fusarium wilt, rust, stemphylium blight, anthracnose, and botrytis gray mold (Taylor et al., 2007).

AB is the major disease problem in many lentil-producing countries, including Australia, Canada, Argentina, Ethiopia, India, New Zealand, and Pakistan (Erskine et al., 1994; Ye et al., 2002). AB in lentil is caused by the ascomycete species *Ascochyta lentis* Vassilievsky. The disease causes lesions on stems, leaves, petioles, and pods. Plant death is common following seedling infection, while infection of mature plants leads to the reduction in vigor, with subsequent decrease in the yield and quality of the seed (Morrall and Sheppard, 1981). Yield losses of up to 40% due to foliar infection have been reported, but the loss of economic value due to seed damage may be more than 70%, as seed can quickly become unsaleable (Gossen and Morrall, 1983, 1984; Brouwer et al., 1985). AB may be controlled through the use of fungicides (Bretag, 1989; Ahmed and Beniwal, 1991), but the most effective, economic, and environmentally sustainable method of control is the development of disease resistant cultivars (Ye et al., 2002).

A number of sources of genetic resistance to AB have been identified (Ahmad et al., 1997; Ford et al., 1999; Nguyen et al., 2001; Ye et al., 2003), including in cultivars such as Indianhead and Northfield (syn. ILL5588) which have been extensively exploited by lentil breeding programs, especially in Australia and Canada (Tar'an et al., 2003). Two independent AB resistance genes, *Ral2* (dominant) and *ral2* (recessive) were identified from Northfield and Indianhead, respectively (Andrahennadi, 1994; Chowdhury et al., 2001). A third (dominant) resistance gene *AbR*_1_, that is active in foliar tissue was also identified in Northfield on the basis of genetic segregation (Tay and Slinkard, 1989) and genetic mapping studies (Ford et al., 1999). Ye et al. (2003) also identified two dominant resistance genes in Northfield, controlling major and moderate resistance, respectively, as well as two additive recessive genes in Indianhead. However, a limited number of disease resistance genes have been placed on lentil genetic linkage maps (Ford et al., 1999; Tar'an et al., 2003). Molecular genetic marker loci such as those generated by random amplified polymorphic DNA (RAPDs) and sequence characterized amplified region (SCARs) systems, were associated with all the known AB resistance genes from Indianhead and Northfield (Ford et al., 1999; Chowdhury et al., 2001; Tar'an et al., 2003).

An additional novel source of resistance, accession ILL7537, exhibits resistance to a number of Australian pathogen groups at a higher level of resistance than either Indianhead or Northfield (Nguyen et al., 2001). The resistance phenotype is thought to be due to at least two dominant resistance genes, distinct to those of Northfield, which were characterized by crosses with susceptible genotypes and subsequent quantitative trait loci (QTL) identification (Rubeena et al., 2006).

Some conflicting results have been obtained from studies of the genetic basis of resistance to infection by *A. lentis*, possibly due to the effects of multiple phenotypic screening methods, variable environmental conditions and variation in the size of evaluated populations (Ford et al., 1999). Pathogen diversity also contributes to the variable assessments of resistance status. Isolates capable of overcoming the dominant resistance gene derived from Northfield are now well-characterized, and have been found in Australia and Canada (Tar'an et al., 2003; Davidson et al., 2016). Given the historical importance in Australia of the formerly resistant cultivar Nipper, of which Northfield and Indianhead are parents, the newer aggressive isolates have been termed “Nipper-virulent” (Davidson et al., 2016). The breakdown in resistance in Nipper also coincided with a reduction in the resistance of a number of Australian cultivars for which resistance was derived from Northfield. However, it has been determined that Nipper does not contain a major resistance gene from Indianhead, unlike a number of other resistant cultivars, suggesting that Indianhead-derived genes are still capable of conferring full resistance against a large proportion of a pathogen population in the field (Davidson et al., 2016). This is also the case in Canada (Albert Vandenberg, pers. comm.), in which AB is an important fungal diseases.

Molecular genetic markers in close linkage with AB resistance genes would permit accelerated development of elite lentil genotypes with resistance to this disease. However, the technologies that have previously been used (Ford et al., 1999; Chowdhury et al., 2001; Tar'an et al., 2003; Rubeena et al., 2006) are not optimal for diagnostic screening in a breeding programme. In addition, previous molecular genetic marker-based maps of lentil have typically been low-density, which limit the capacity to identify marker loci in sufficiently close linkage. However, a number of transcriptome sequencing studies for lentil have generated expressed sequence tag (EST) databases, delivering large numbers of EST-derived (and hence gene-associated) simple sequence repeat (SSR) and single nucleotide polymorphisms (SNP) markers (Kaur et al., 2011, 2014; Sharpe et al., 2013). These marker systems have been used to construct dense genetic linkage maps, and to identify QTLs (Sharpe et al., 2013; Kaur et al., 2014). Sequence-linked genetic markers also facilitate the identification of bridging loci between population-specific genetic maps, and subsequent integration to produce high-density consensus structures (Sudheesh et al., 2015a,b).

The present study describes the construction of genetic maps for three populations derived from pair-wise combinations of the lentil varieties Indianhead, Northfield, and Digger. Although partial breakdown of the Northfield-type AB resistance has occurred (Davidson et al., 2016), QTLs for the effective Indianhead-type resistance were identified. The predictive capacity of markers linked to AB resistance genes was also tested using a diverse germplasm collection, or “validation panel.” The population-specific maps were integrated to form a consensus structure suitable for application in lentil molecular breeding.

## Materials and methods

### Plant materials

Two segregating genetic mapping populations were developed from crosses between single genotypes of Indianhead (resistant to AB) with Northfield (previously resistant to AB) and Digger (moderately resistant to AB), respectively. The third genetic mapping population was developed by crossing single genotypes from Northfield and Digger. All three populations were initiated at DEDJTR-Horsham in 2002, based on single seed descent from F_2_ progeny for four generations in the glasshouse to generate the following F_6_ recombinant inbred line (RILs): Indianhead × Northfield [IH × NF] – 117 RILs; Indianhead × Digger [IH × DIG] – 112 RILs; and Northfield × Digger [NF × DIG] – 114 RILs.

A germplasm panel composed of a set of 79 diverse lentil genotypes was used for validation of AB resistance-linked markers. The panel included Australian lentil cultivars, varieties, and breeding germplasm, along with international lentil germplasm from the International Center for Agricultural Research in the Dry Areas (ICARDA) and North American breeding programs (see Supplementary Table 1 for list).

Plants were grown in glasshouse at 20 ± 2°C under a 16/8 h light/dark photoperiod regime. Genomic DNA was extracted from young leaves using the DNeasy® 96 Plant Kit (QIAGEN, Hilden, Germany) according to the manufacturer's instructions. Approximately 6–8 leaflets per sample were used for each extraction, and were ground using a Mixer Mill 300 (Retsch®, Haan, Germany). DNA was resuspended in milliQ water to a concentration of 50 ng/μl and stored at −20°C until further use.

### SSR and SNP genotyping and genetic linkage mapping

Genomic DNA-derived (Hamwieh et al., 2005) and EST-derived SSRs (Kaur et al., 2011) were screened on the mapping parents for polymorphism detection, and the resulting polymorphic markers were screened on the RILs as described previously (Schuelke, 2000; Kaur et al., 2014). For SNP genotyping, a previously described set of 768-plex SNPs (Kaur et al., 2014), was selected and genotyped using the GoldenGate™ oligonucleotide pooled assay (OPA; Illumina Inc., San Diego, USA). Genetic linkage mapping and visualization of the linkage groups (LGs) of RILs were performed as described previously (Kaur et al., 2014). All sequences underlying mapped SNP markers from the present study were analyzed with BLASTN against the equivalent sequences of Sharpe et al. (2013) and the *Medicago truncatula* genome (Mt4.0) at a threshold E-value of 10^−10^. This information was used to assign identity and orientation to the lentil LGs. Visual comparisons between genetic linkage maps were performed using the Strudel software package (Bayer et al., 2011).

### Consensus linkage map construction

The genetic maps from the present study were combined with the Cassab × ILL2024 map of Kaur et al. (2014), which shared a high proportion of common markers, to generate a consensus map using MergeMap (Wu et al., 2011). Each LG from the consensus linkage map was visualized using MapChart (Voorrips, 2002). The visual comparison of the consensus map with individual RIL-based maps was performed using the Strudel software package (Bayer et al., 2011).

A comparative analysis of this consensus map was made to the sequence-based map of Sharpe et al. (2013) as well as the pseudomolecules of *M. truncatula*, using BLASTN analysis of sequences underlying mapped SNP markers. Dot-plots of the comparison of the consensus linkage map and *M. truncatula* pseudomolecules were generated using the R software package with the xyplot function from the Lattice CRAN library (Sarkar, 2014).

### Phenotypic assessment of AB resistance

Resistance to AB was assessed for the three RIL mapping populations in four separate experiments (single experiment for each of IH × NF and IH × DIG, two experiments for NF × DIG). A total of 3–6 seeds from each RIL and respective parents were sown into individual pots (8 × 18 × 6.5 cm), filled with Van Schaik's Bio Gro (Bio Gro Pty. Ltd., Victoria, Australia) pine bark potting mix. The potting mix consisted of 1000 L of composted pine bark (Bio Gro), 1 kg Floranid® N32 (Compo, Münster, Germany), 1 kg 8–9 month Osmocote® (Scotts, NSW, Australia), 1 kg 3–4 month Osmocote® (Scotts), 225 g micronutrients MicroMax® Complete (Scotts), 225 g SP Quality® FeEDDHA Chelate (6% Fe; Libfer-BASF, Victoria, Australia), 30 kg agricultural lime (Sibelco, Victoria, Australia), and 2 kg Saturaid® (Debco, NSW, Australia). After sowing, the pots were placed in a controlled environment room (CER) at 15°C, under a 12/12 h light/dark cycle regime in four plastic tents (160 × 80 × 80 cm) in a randomized complete block design, with one replicate per tent. Pots were watered by hand as required. Seedlings were inoculated 2 weeks after sowing, as described below.

Three different isolates of *A. lentis* (Supplementary Table 2) were used, the Australian reference isolate AL4 being applied to NF × DIG, isolate FT13038 to IH × NF, and isolate FT12013 to IH × DIG. Parents of the mapping populations exhibit different level of resistance to *A. lentis* isolates, hence isolates which could most effectively distinguish between the parents were selected for application on mapping populations. AB resistance-specific screening of the germplasm panel was conducted using FT12013, which has been isolated recently and is known to have overcome the resistance of at least one of the resistance (R) genes present in cultivars Northfield and Nipper (Davidson et al., 2016). A sub-set of germplasm panel lines were further evaluated using multiple pathogen isolates (Supplementary Table 2).

To initiate fungal cultures, mycelial plugs of *A. lentis* isolates were transferred from storage vials to potato dextrose agar (PDA) in 9 cm diameter culture dishes and incubated for 14 days under fluorescent light and near ultraviolet light under a 12/12 h light/dark cycle regime at room temperature. A 1 L conidial suspension of the isolate was prepared by flooding the plates with sterile reverse osmosis (RO) water and gently rubbing the culture surface with a sterile glass rod to suspend the conidia. The concentration was determined with the aid of a hemocytometer, adjusted to 1 × 10^6^ conidia mL^−1^ and the surfactant Tween 20 [0.02% (v/v)] was added. The conidial suspension was sprayed onto the seedlings until run-off occurred. Each tent had two ultrasonic humidifiers, one at either end, using RO water to maintain leaf wetness. The two ultrasonic humidifiers within each tent were switched on immediately after inoculation for 1 h and every day thereafter for 1 h to promote lesion development which continued until disease assessment could be performed. Disease incidence was assessed for each seedling 14 days after inoculation as percentage area of plant diseased (% APD), incorporating leaf and stem lesions (Davidson et al., 2016).

Data was analyzed to estimate genotype-specific adjusted means for any spatial effects using residual maximum likelihood (REML) in Genstat v14.1 (Lane et al., 2011). Means of % APD data from each trial were used to construct frequency distribution histograms.

### QTL analysis and identification of sequences associated with flanking genetic markers

QTL detection was performed using marker regression, simple interval mapping (SIM) and composite interval mapping (CIM) in QTL Cartographer v 2.5 (Wang et al., 2012). For SIM, an arbitrary LOD threshold of 2.5 was used to determine significance, while for CIM, significance levels for LOD thresholds were determined using 1000 permutations. SIM and CIM analysis of the NF × DIG mapping population for both experimental treatments failed to identify any QTL associated with AB resistance. Data for this population was consequently not considered further for trait-dissection purposes, but was used for consensus linkage map construction.

### Genotyping of the diverse germplasm panel

Genetic markers flanking AB resistance QTL-containing intervals from the IH × NF and IH × DIG mapping populations were used for genotypic analysis. SSR primer synthesis and PCR amplifications were performed as described above. SNP genotyping was performed using KASP™ genotyping chemistry (LGC, Middlesex, UK) as described in Javid et al. (2015).

## Results

### Polymorphic markers for map construction

A total of 546 publicly available SSR markers (30 genomic DNA-derived SSRs and 516 EST-SSRs) were screened for polymorphism detection in the mapping populations. Of the former, up to 87% detected polymorphisms, while relatively smaller proportions of the EST-SSR markers were polymorphic (Table 1). After the χ^2^ analysis (*P* < 0.05), final sets containing a maximum of 61 (IH × NF) and a minimum of 31 (IH × DIG) segregating SSR markers were used for linkage mapping.

**Table 1:** **Total number of markers analyzed, tested for polymorphism, and assigned to genetic linkage map locations**.

**Marker type**	**Total number of markers screened**	**Polymorphic markers**			**Markers used for linkage mapping**			**Mapped markers**		
		**IH × NF**	**IH × DIG**	**NF × DIG**	**IH × NF**	**IH × DIG**	**NF × DIG**	**IH × NF**	**IH × DIG**	**NF × DIG**
Genomic DNA-derived SSR	30	26	19	22	17	9	22	11	7	16
EST SSR	516	45	42	35	44	22	35	31	22	28
SNP	768	435	329	328	422	315	310	418	300	286
Total markers	1314	577	451	442	483	346	367	460	329	330

A commonly used set of 768 SNPs was screened on the mapping populations, of which 328 (NF × DIG) to 435 (IH × NF) detected polymorphism (Table 1). A small number (24) of polymorphic loci were shared between all three mapping populations, but up to 490 loci were common between any two populations. After the χ^2^ analysis (*P* < 0.05), SNP markers that did not segregate in accordance with the expected Mendelian inheritance ratio were excluded, which resulted in a final set of up to 422 SNP markers (IH × NF; Table 1).

### Genetic linkage mapping

A total of 483 loci (IH × NF), 346 loci (IH × DIG), and 367 loci (NF × DIG) were used for linkage mapping (Table 1). Details of the number of assigned LGs, markers, and the cumulative length of maps are provided in Table 2 and Supplementary Table 3. The proportion of loci assigned to LGs was 95.3, 95.1, and 89.9% for the IH × NF, IH × DIG, and NF × DIG maps, respectively, while the remaining markers were unlinked. The IH × NF map contained a higher number of markers with lower average marker density than the other two maps. The distribution of markers was not uniform across the LGs, as some regions of high and low marker density were observed. Significant commonality of marker order was observed between the three maps, although distances were not always in similar proportion (Supplementary Figures 1A–C). Some markers (52 in total) were assigned to different LGs on the various maps. One such major anomaly was observed for the IH × DIG and NF × DIG maps, in which 49 markers (45 SNPs and 4 SSRs) were located in a segment on LG4, while for the IH × NF map, the corresponding positions of those markers were on LG6 (Supplementary Figure 1D). Sequence similarity searches against the *M. truncatula* genome of DNA sequences underlying those SNP loci revealed matches to MtChr7 (Supplementary Table 4), which displays macrosynteny with lentil LG6.

**Table 2:** **Marker distribution over the LGs of IH × NF, IH × DIG, and NF × DIG genetic linkage maps**.

**Linkage group**	**Map length (cM)**			**Number of mapped marker**			**Average marker density**		
	**IH × NF**	**IH × DIG**	**NF × DIG**	**IH × NF**	**IH × DIG**	**NF × DIG**	**IH × NF**	**IH × DIG**	**NF × DIG**
LG1	199.1	143.4	74.1	44	32	8	4.5	4.5	9.3
LG1.2		18.8	182.3		7	29		2.7	6.3
LG2	195.7	87.9	126.3	84	29	21	2.3	3.0	6.0
LG2.2	13.5	211.5	155.3	3	35	30	4.5	6.0	5.2
LG3	151.7	245.5	163.8	53	65	26	2.9	3.8	6.3
LG3.2	31.9	14	29	12	3	7	2.7	4.7	4.1
LG3.3			73.9			7			10.6
LG3.4			11.3			5			2.3
LG4	310.2	205.7	431.6	80	78	88	3.9	2.6	4.9
LG5	187.3	197.2	310	58	38	46	3.2	5.2	6.7
LG6	190.4	22.8	38.8	69	4	6	2.8	5.7	6.5
LG7	176.3	13.2	280	54	6	52	3.3	2.2	5.4
LG7.2	5.5	40.5	37.7	3	8	5	1.8	5.1	7.5
LG7.3		102			24			4.3	
	1461.6	1302.5	1914.1	460	329	330	3.2	4.1	6.2

BLASTN analysis of the DNA sequences corresponding to 163 map-assigned SNPs detected significant similarity matches to 102 sequences assigned to the genetic map of Sharpe et al. (2013) (Supplementary Table 4). This analysis supported establishment of bridging loci between six LGs (LG1, 2, 3, 4, 5, and 7), although no common markers could be identified for LG6 (Table 3). Marker order was generally co-linear between the two studies, although minor discrepancies were observed for some markers.

**Table 3:** **Marker distribution over the LGs of the consensus linkage map**.

**Linkage group**	**Predicted Mt chromosome**	**LG from Sharpe et al. (2013)**	**Number of mapped markers**	**Map length (cM)**	**Average marker density**
LG1	1/5	1	79	332.9	4.2
LG2	2/6	2	131	429.7	3.3
LG3	3	3	110	353.0	3.2
LG4	4/7/8	4	117	398.3	3.4
LG5	5/1	5	94	403.1	4.3
LG6	7		72	192.7	2.7
LG7	4/8	7	86	319.9	3.7
Total			689	2429.6	3.5

### Consensus linkage map construction

**Figure 1:**
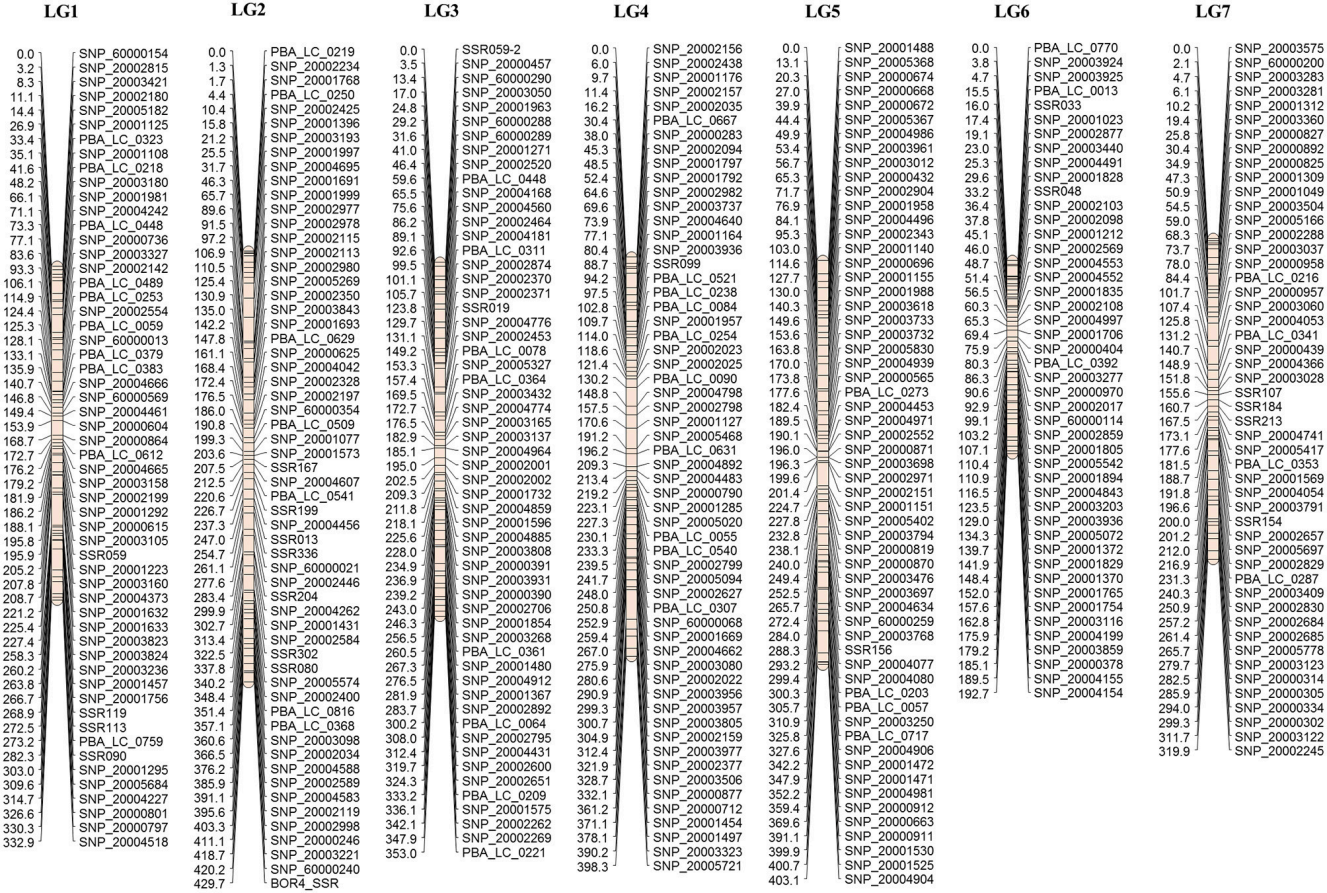
**Consensus map of lentil, with marker loci shown on the right of LGs, and distances between markers indicated in cM on the left**. For presentation purposes, only one of a set of co-located genetic markers is shown on the map.

Data from the mapping populations described in the present study and a previously published mapping population (Cassab × ILL2024, containing 318 markers) was used to construct the consensus linkage map of lentil. The common markers on homologous LGs from the RIL-based maps served as bridges for integration into a consensus structure. A total of 149 markers were unique to single populations (62 – IH × NF; 15 – IH × DIG; 34 – NF × DIG; 38 – Cassab × ILL2024), the remainder acting as bridging loci between two or more maps. As the SNP marker sets were selected to obtain a large number of polymorphic markers for all populations under study, only a small number of markers (18) were common across all four RIL-based maps. The largest number of common markers (113) was between the IH × NF – IH × DIG maps, followed by the IH × NF – NF × DIG (92) and NF × DIG – Cassab × ILL2024 (17) comparisons. The 52 markers that did not display consistent LG assignment were excluded. IH × NF linkage map was used as the skeleton map as that map contained the highest number of markers, with lower average marker density than other three maps. Also, the IH × NF linkage map revealed a high degree of colinearity of marker order when compared to previously published lentil maps (Sharpe et al., 2013; Kaur et al., 2014), as well as a superior level of conserved synteny with the genome of *M. truncatula*. Finally, 689 marker loci (94 SSRs and 595 SNPs) were assembled into seven LGs (Figure 1, Table 3, Supplementary Table 5), with a total length of 2429.6 cM, lengths of LGs varying from 192.7 cM (LG6) to 429.7 cM (LG2), with an average density of one marker per 3.5 cM. The marker order of consensus map was largely colinear between the individual RIL-based maps, although several inversions and local rearrangements were observed (Supplementary Figures 2A–C).

**Figure 2:**
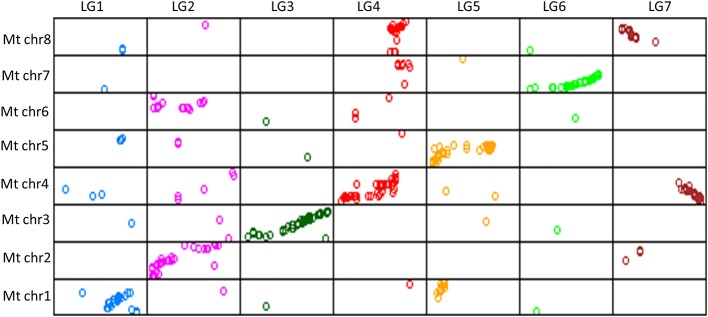
**Dot-plot representing correspondence between lentil consensus map linkage groups 1 through 7 (top) and *M. truncatula* chromosomes 1 through 8 (left side)**.

Of the 689 markers assigned to the seven LGs of the lentil consensus map, 522 (76%) identified orthologous sequences on the eight *M. truncatula* chromosomes, with a minimum of 62% (LG1) and a maximum of 88% (LG6). The relative correspondences and orientations of consensus map LGs and *M. truncatula* pseudomolecules were determined by examining dot-plots, which showed large segments of conserved macrosynteny, as anticipated (Figure 2). LGs 3 and 6 were relatively colinear along their entire length with pseudomolecules 3 and 7. Comparative analysis also indicated that some genome rearrangements have occurred in lentil. For example, LG2 exhibited macrosynteny with pseudomolecules 2 and 6 (Figure 2), and major evolutionary translocations were observed for pseudomolecules 1 and 5 relative to LG1 and 5 of lentil. LG7 showed significant matches to positions on pseudomolecules 4 and 8, while LG4 showed similarity to genomic regions on pseudomolecules 4, 7, and 8.

### Phenotypic analysis of RIL populations and QTL detection

Significant differences in plant symptom scores (%) for parents and RILs of each mapping population were observed following infection with *A. lentis* isolates. Severity of AB infection varied significantly for the IH × NF mapping population, scores ranging from 0 to 30%. The Indianhead and Northfield parents showed 0 and 12% infection, respectively, while a small proportion of RILs displayed transgressive segregation toward scores characteristic of higher susceptibility than Northfield. Similar effects were observed for the IH × DIG mapping population (Indianhead, 0%; Digger, 5%; RILs, 0–30%; Supplementary Figure 3).

**Figure 3:**
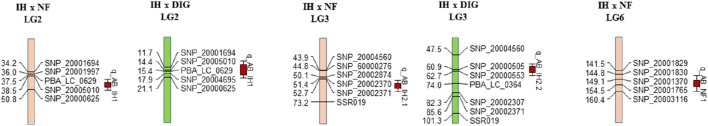
**Localization of QTLs associated with resistance to ***A. lentis*** on the IH × NF-derived genetic map and IH × DIG-derived genetic map**. The name is provided at the top of each LG. Distances of the loci (cM) are shown to the left and names of loci are shown to the right side of LGs. For presentation purposes, only selected markers are shown on the map.

For IH × NF, CIM analysis detected three QTLs (AB_IH1, AB_IH2.1, and AB_NF1) associated with AB resistance, on LG2, LG3, and LG6, explaining c. 47, 15, and 7% of the phenotypic variance (*V*_*p*_), respectively (Figure 3, Table 4). For AB_NF1, the resistance determinant was derived from Northfield, while the other two QTL regions were associated with the resistant parent, Indianhead. For IH × DIG, CIM detected two QTLs from IH (AB_IH1 and AB_IH2.2), which were at the same locations (LG2 and LG3) as those identified in the IH × NF mapping population, accounting for c. 30 and 22% of *V*_*p*_, respectively (Figure 3, Table 4). The LOD peak of AB_IH1 coincided with the markers PBA_LC_0629 and SNP_20005010 for both mapping populations, while the markers flanking AB_IH2.2 (SNP_20000505 and SNP_20000553) were not variant on the IH × NF map. However, the presence of common markers between these two maps in the vicinity of the QTL-containing region supports the inference that a common genomic region on LG3 is responsible for conferring resistance.

**Table 4:** **Identification of QTLs for AB resistance on IH × NF and IH × DIG genetic maps based on CIM**.

**Mapping population**	**QTL name**	**Linkage group**	**Flanking markers**	**Position (cM)**	**LOD threshold**	**Maximum LOD score**	**% Phenotypic variance**	**Additive effect**
IH × NF	AB_IH1	LG2	PBA_LC_0629	37.5–38.5	3.1	16.8	47	3.9174
			SNP_20005010					
	AB_IH2.1	LG3	SNP_20002370	51.4–52.7	3.1	7	15	2.2097
			SNP_20002371					
	AB_NF1	LG6	SNP_20001370	149.1–154.5	3.1	3.5	7	–1.4697
			SNP_20001765					
IH × DIG	AB_IH1	LG2	SNP_20005010	14.4–17.9	3.1	13.1	30	3.7020
			PBA_LC_0629					
			SNP_20004695					
	AB_IH2.2	LG3	SNP_20000505	60.9–62.7	3.1	9.7	22	3.2251
			SNP_20000553					

### Phenotypic and genotypic analysis of diverse germplasm panel

Responses of the germplasm panel members to inoculation treatments with *A. lentis* isolate FT12013 were consistent. Severity of AB infection varied significantly, with scores from 0 (no symptoms) to a highest score of 25% APD. Approximately half of the panel lines, including those with a known high level of resistance (cultivars Indianhead, CDC Matador and PBA Ace) showed no foliar infection symptoms, while the majority of the remaining lines displayed intermediate scores (5–18%). A total of four lines, including cultivars Cumra and PBA Flash, showed a susceptible reaction to AB, with foliar infection levels of 20–25% (Supplementary Figure 4).

The allelic identity for genetic markers linked to AB resistance QTLs was highly correlated with the phenotypic assessment data, and clearly distinguished between resistant, moderately resistant, and susceptible genotypes. As the largest proportion of phenotypic variance (47%) was explained by AB_IH1, precedence was given to allelic identity data for markers linked to that QTL. Of the 79 genotypes tested, marker allele predictions were accurate for 68 (86%). For those lines where the phenotype and genotype data were discordant, six contained the resistance allele but were susceptible (false-positive), while five were resistant but only contained the moderate resistance-associated allele from Digger (false-negative; Supplementary Table 6). The 11 anomalous genotypes were further examined using pedigree information, revealing that all false-positive genotypes lacked the RIL parental lines in their pedigrees.

A detailed analysis of phenotype-genotype data correlations for the sub-set of panel lines that were infected with multiple Australian *A. lentis* isolates is provided in Table 5. The SNP marker SNP20005010 was found to most reliably predict the presence of the resistance allele for AB_IH1. For AB_NF1, SNP 20001370, and SNP20001765 were found to be associated with a minor gene that appeared to confer partial resistance to a FT14125 isolate (2014 isolate from Horsham, Victoria). For AB_IH2.1, the marker identified in the IH × NF population, SNP20002370, provided a prediction of partial resistance to isolates such as FT14125. Markers associated with AB_IH2.2 were apparently not correlated with resistance to *A. lentis* isolates.

**Table 5:** **Phenotypic scores of AB infection on lentil germplasm panel inoculated with ***A. lentis*** isolates alongside genotyped markers for three QTL regions**.

**Lentil line**	**Pedigree**	**AB field rating**	**Phenotypic scores of AB infection on lentils inoculated with five separate** ***A. lentis*** **isolates in controlled environment**					**AB_IH1**	**AB_IH2.1**	**AB_NF1**
			**Historical isolate, Kewell**	**FT14125**	**FT10002**	**FT12013**	**FT13013**	**SNP_20005010**	**SNP_20002370**	**SNP_20001370**
ILL7537		R	0	0		0.6	0	+	+	−
Indianhead		R	3.7	0		0	0	+	+	−
CIPAL1504	PBAACE/PBABOLT	MR	10.4	1.1		0	0.1	+	+	+
CIPAL1522	PBAACE/04-299L-05HG1001-05HSHI2006	R	4.5	0.2		0	0	+	+	+
PBA Ace	CIPAL0501/96-047L*99R099	R	8.7	1.9	0	0	0	+	+	+
PBA Herald XT	96-047L*99R060-EMS02	R	2.5	0.9	0	0	0	+	+	+
PBA Jumbo2	CIPAL0205/BOOMER//CIPAL0401	R	0	0	0	0.6	0	+	−	−
CIPAL1501	PBABOLT*06G1001/CIPAL0804	R	0	0.4			0.1	+	−	+
CIPAL1301	PBABOLT/02-325*03HS001	R	0.1	0.6	0	0	0.6	+	−	+
CIPAL1502	PBABOLT*06G1002/CIPAL0804	R	0.3	0.1			0	+	−	+
CIPAL1523	PBABLITZ/04-190L-05HG1001-05HSHI2001	MR	5.7	5.1		0	0	+	−	+
PBA Bolt	ILL7685/96-047L*99R060	MR	5.9	4.6	0	0	0	+	−	+
CIPAL1521	PBABOLT/04-299L-05HG1001-05HSHI2006	R	7.3	2.4		1.3	0	+	−	+
PBA Hurricane XT	PBAFLASH/96-047L*99R060M3	MR	12.8	4.7	0.1	0	1.1	+	−	−
CIPAL1422	PBAHERALD/PBABOLT	R	16.0	3.4	0	0	0.7	+	−	+
Boomer	DIGGER/PALOUSE	MR	0.9	0.1	0.1	0.6	3.0	−	+	−
Nipper	INDIANHEAD/NORTHFIELD//NORTHFIELD	MR/MS	0.3	0	0.1	9.4	10.6	−	+	−
PBA Giant	PBAFLASH/BOOMER	MR/MS	3.4	1.3	1.3	2.6	4.7	−	+	+
Nugget	NORTHFIELD/ILL5714	MR/MS	3.6	3.0	4.7	7.5	4.3	−	+	+
Northfield		MR/MS	0	0.1	3.7	8.1	5.6	−	−	+
PBA Flash	ILL7685/NUGGET	MS	3.1	2.0	4.6	16.9	5.8	−	−	+
PBA Blitz	CUMRA/INDIANHEAD//CASSAB	MR/MS	1.8	0.2	3.6	5.6	3.9	−	−	−
PBA Greenfield	CIPAL0205/BOOMER//PBAFLASH	MR/MS	0	0	3.6	14.4	6.1	−	−	−
PBA Jumbo	ALDINGA/CDCMATADOR	MR/MS	1.0	0	3.3	15.6	9.4	−	−	−
Cumra		S	22.9	3.1		14.4	8.0	−	−	−

*+, Preferred (resistance) allele present; −, Non-preferred (susceptible) allele present*.

## Discussion

### Genetic linkage mapping

A substantially lower proportion of EST-SSR markers detected polymorphism (10%) as compared to genomic derived-SSRs (87%), as previously reported for the same marker set (Kaur et al., 2014). SNP genotyping revealed a total of 583 markers (75%) as polymorphic, but only a small number were found to be common between the three RIL populations. The 768-plex SNP assays used in this study were developed from a range of cultivated genotypes (including the parental genotypes of the mapping populations) and further selected to maximize the number of population-specific SNPs (Kaur et al., 2014), accounting for the observed variable proportions of polymorphic loci and limited commonality between populations.

A number of genetic linkage maps have recently been developed for lentil, through the use of SSR and SNP marker technologies (Sharpe et al., 2013; Gujaria-Verma et al., 2014; Kaur et al., 2014). The cumulative lengths of maps from this study were marginally higher than those from previous studies (Sharpe et al., 2013; Kaur et al., 2014), possibly due to the effects of a higher number of map-assigned markers, or the genetic constitution of mapping populations (potentially influencing rates of recombination). The distribution and order of markers across LGs in each genetic linkage map were highly comparable, except for those markers anomalously assigned to LG4 in individual RIL-based maps, but confirmed to be located on LG6 on the basis of known macrosynteny with MtChr7. This discrepancy could be due to chromosomal rearrangement events in specific genotypes, but may also be attributable to paralogous sequence effects. As the EST-derived markers may have been derived from individual members of a gene family, an assay designed to detect a polymorphism between two contrasted genotypes in one gene copy (but with no variation in a second gene copy) may inadvertently detect the reverse situation between a second pair of contrasted genotypes, thus generating the appearance of a re-located marker locus (Schwarz-Sommer et al., 2003; Somers et al., 2004). Identification of multiple loci of this nature may reflect the presence of ancestral segmental duplication events, which are known to have been common during the evolution of the Fabaceae.

Merger of the four RIL-based maps through use of common genetic markers generated a consensus map containing a total of 689 markers, higher than any previously constructed population-specific map for lentil (Sharpe et al., 2013; Kaur et al., 2014). Comparative analysis also supported identification of bridging loci between six LGs of the consensus map with that of Sharpe et al. (2013), but the two maps could not be integrated into a single structure due to insufficient common markers, especially for LG6 which is devoid of such markers.

Legumes display extensive conservation of gene order, even between species which differ dramatically in terms of genome size (Choi et al., 2004; Phan et al., 2006). In the present study, comparative analysis was performed between the *M. truncatula* genome and the gene sequences associated with markers assigned on the consensus map. As previously reported (Sharpe et al., 2013; Gujaria-Verma et al., 2014; Kaur et al., 2014), direct and simple correspondences are observed between *M. truncatula* pseudomolecules and lentil LGs, although some evolutionary translocations and non-colinear relationships were also detected.

Although draft or complete genome sequences for many plant species have been made available [e.g., *M. truncatula* (http://www.medicago.org), chickpea (*Cicer arietinum*); Varshney et al., 2013), soybean (*Glycine max*; Schmutz et al., 2010), and pigeon pea (*Cajanus cajan*; Varshney et al., 2011), crop improvement programs based on recombinational assortment of favorable gene variants require the construction of genetic maps. Moreover, whole genome assemblies require high-density linkage maps to assist assembly and assess the quality of sequenced genomes. An international effort to deliver a reference lentil genome sequence is currently underway, leading to the recent release of an initial draft assembly from the cultivar CDC Redberry. However, this assembly is still in a preliminary form, with minimal gene annotation and limited access (Bett et al., 2016). The consensus map generated from the current study could potentially help to further improve the current draft lentil genome.

### Identification of QTLs and validation of linked genetic markers

Multiple studies have been conducted in order to identify superior sources of resistance to AB in lentil, corresponding to genes of major effect (Ahmad et al., 1997; Ford et al., 1999; Nguyen et al., 2001). The results of the present study are consistent with previous studies (Rubeena et al., 2006; Gupta et al., 2012) that demonstrated the presence of multiple genes for AB resistance with different modes of action in different lentil genotypes. The identification of common QTLs (AB_IH1, AB_IH2.1/AB_IH2.2) between mapping populations with a shared parent (Indianhead) provides confidence in the process of QTL identification. Differences observed between the *V*_*p*_ proportions accounted for the common QTLs could be due to variability between conditions of the two screening experiments, or the influence of partial resistance genes contributed by Northfield and Digger.

In the context of a fungal pathogen population that is able to overcome plant resistance genes, the properties of the three QTLs identified in this study largely explain the observed genetic resistance to two alternative AB pathotypes recently isolated from field-grown crops. The QTL of largest effect (AB_IH1, identified in both IH × NF and IH × DIG mapping populations) accounted for the majority of AB resistance when using the current, most aggressive, field-derived isolates (Davidson et al., 2016). The Indianhead-derived QTL allele conferred resistance to these isolates, of which FT12013 was a representative. Recombination in the vicinity of this QTL appears to have occurred in Australian germplasm such as cultivar Nipper, which contains the allele of the flanking SSR marker PBA_LC_0629 characteristic of Indianhead (which was a parent of Nipper), but not the corresponding allele at the coincident SNP marker (SNP20005010). Nipper, and others with the same genotype (such as PBA Greenfield) do not have resistance to isolate FT12013, probably indicating that the candidate R gene is closer to the SNP than the SSR marker. Allelic identity at AB_IH1 was found to be predictive of resistance to the aggressive “Nipper-virulent” isolate (FT12013) in the majority of Australian lentil germplasm testing in the panel. However, this relationship was not conserved for all diverse germplasm, such as ICARDA lines ILL2024 and ILL6788 (two parental lines that have used in the Australian lentil breeding program), which were susceptible to FT12013. As a consequence, resistance status was not predictable for cultivar PBA Bounty, which was derived from selected progeny of a cross with ILL6788.

In 2014, a field isolate (FT14125) with a different pattern of pathogenicity on lentil genotypes was identified from a population of *A. lentis* at Horsham, Victoria. This isolate is hence thought to belong to a pathotype grouping differing from the currently dominant field isolates that has overcome the resistance derived from Northfield (which is also found in cultivars Digger and Nugget). The Indianhead-derived allele at locus SNP20005010 was not found to be necessary for resistance to FT14125, and cultivars Nipper, PBA Blitz, PBA Jumbo, and PBA Greenfield (which are susceptible to Nipper-virulent isolates) exhibited complete resistance to this isolate in a controlled environment trial having only the Digger-derived allele at SNP20005010 and the Indianhead-derived allele at AB_NF1 in common. The Indianhead-derived allele at AB_IH2.1 (SNP_20002370) also appeared to confer partial resistance to this isolate in the absence of the previous two alleles (e.g., for PBA Ace, PBA Herald XT, and CIPAL1522).

There is also evidence for AB resistance genes apart from the three identified QTLs. Northfield and Boomer demonstrate greater resistance to AB than expected on the basis of QTL-associated genotype. Northfield has the same genotype as the susceptible cultivar PBA Flash (lacking Indianhead-derived alleles), and a similar susceptibility to “Nipper-virulent” isolates (such as FT12013), but is at present significantly more resistant than PBA Flash, in the field environment and to isolate FT14125. Similarly, Boomer lacks the Indianhead-derived allele at SNP20005010, but displays moderate resistance to field isolates of AB, significantly higher than for cultivars such as Nipper and Nugget (which have a similar genotype at the three identified QTLs).

A direct comparison of QTL-flanking loci identified in the current study with those from previous studies (Rubeena et al., 2006; Gupta et al., 2012) could not be performed, due to the lack of common markers. Furthermore, previous LG nomenclature differed from that used in more recent studies (Sharpe et al., 2013). However, AB_NF1 on LG6 is comparable in location to a previously described QTL (QTL5 on LG1—Rubeena et al., 2006, QTL1 on LG1—Gupta et al., 2012), based on a common SSR locus location. Moreover, the various mapping populations in these studies were related through the common parent Northfield, which conferred seedling-based AB resistance. A previous study (Chowdhury et al., 2001) reported the development of two SCAR markers from RAPD markers linked to the *ral2* (UBC227_1290_ and OPD-10_870_) gene. However, that study revealed that SCAR marker developed from UBC227_1290_ was monomorphic, and the other SCAR marker developed from OPD-10_870_ was not efficient in discriminating different phenotypes among F_2_ progeny (Chowdhury et al., 2001), and so was not screened in the present study.

The markers identified in the present study will be highly useful for deployment of desirable R genes into a lentil breeding program, allowing pyramiding with other effective genes to confer durable resistance. The current data suggests that AB_IH1 confers the highest level of field resistance, but may be enhanced by the presence of AB_IH2.1, while the value of AB_NF1 from the Northfield background has been mostly non-conclusive. Different R alleles from these QTLs have been noted to respond differently to various *A. lentis* isolates, and so further in-depth knowledge of the population structure of pathogen may be required to understand the effects of AB_NF1 on AB resistance. An immediate use of the identified markers will therefore be to select for QTL combinations capable of matching the resistance profile of Indianhead.

As has been recently demonstrated (Davidson et al., 2016), the *A. lentis* pathogen is capable of adaptation to overcome R genes deployed in lentil germplasm. For this reason, continuous surveillance of resistance status is necessary, including analysis of other structured genetic populations in order to locate for AB resistance coming in germplasm such as Boomer and ILL7537, as well as the partial resistance genes present in Northfield and Digger.

In conclusion, the present study has developed valuable genetic resources including RIL-based maps and a consensus linkage map, which will collectively assist other trait-dissection studies for future lentil breeding activities. Evaluation of AB resistance under controlled conditions permitted identification of three and two QTLs in the IH × NF and IH × DIG mapping populations, respectively. Common genomic regions (AB_IH1 and AB_IH2.1/AB_IH2.2) were identified as responsible for AB resistance in both mapping populations, and were associated with the resistant parent, Indianhead while the third genomic region was associated with Northfield parent. Validation of flanking markers across a diverse germplasm demonstrated that these markers predicted the phenotypic responses with high levels of accuracy. The tightly linked molecular markers for AB resistance will enable marker-assisted selection (MAS) of AB resistant cultivars, based on introgression of QTL-containing genomic regions from donor to recipient germplasm.

## Author contributions

SS performed map construction, QTL analysis, marker validation, and contributed to drafting the manuscript. MR contributed to data interpretation and assisted in drafting the manuscript. JD performed the phenotyping of the mapping populations and validation panel and contributed to data interpretation. MJ assisted in performing phenotyping of validation panel. AS performed the genotyping of the mapping populations. JF, ATS, and NC co-conceptualized the project and assisted in drafting the manuscript. SK co-conceptualized and coordinated the project and assisted in drafting the manuscript. All authors read and approved the final manuscript.

## Funding

This work was supported by funding from the Victorian Department of Economic Development, Jobs, Transport and Resources, Australia and the Grains Research and Development Council, Australia.

### Conflict of interest statement

The authors declare that the research was conducted in the absence of any commercial or financial relationships that could be construed as a potential conflict of interest.

## Supplementary material

Supplementary Figure 1 **Comparison between linkage groups of IH × NF, IH × DIG, and NF × DIG maps**. This file shows visual representation of the comparison of **(A)** all LGs in three different maps and the common marker loci between them, **(B)** LG5 from three maps and the common marker loci between them, **(C)** LG7 from three maps and the common marker loci between them, **(D)** LG6 from three maps and the common marker loci between them. White lines represent the corresponding positions of common markers.

Supplementary Figure 2 **Comparison between linkage groups of IH × NF, IH × DIG maps with consensus linkage map**. This file shows visual representation of the comparison of **(A)** all LGs in IH × NF and IH × DIG maps with consensus map and the common marker loci between them, **(B)** LG5 from three maps and the common marker loci between them, **(C)** LG6 from three maps and the common marker loci between them. White lines represent the corresponding positions of common markers.

Supplementary Figure 3 **Frequency distribution histogram**. This file contains frequency histograms generated from AB resistance scores in IH × NF and IH × DIG mapping populations.

Supplementary Figure 4 **Frequency distribution histogram**. This file contains frequency histograms generated from AB resistance scores from lentil germplasm lines.

Supplementary Table 1 **List of validation panel lines**. The table provides the list of lentil validation panel lines used for marker validation with their respective germplasm category.

Supplementary Table 2 **List of *A. lentis* isolate used for phenotyping**. This file contain details of different *A. lentis* isolates used for phenotyping lentil mapping populations and the validation panel.

Supplementary Table 3 **Linkage map statistics from IH × NF, IH × DIG, and NF × DIG maps**. This file contains details of different markers (SSRs and SNPs) and their corresponding positions on different LGs.

Supplementary Table 4 **BLASTN results of the markers**. The data represents the BLASTN-based sequence analysis of DNA sequences underlying SNP markers assigned to three maps against *M. truncatula* genome (Mt4.0) and DNA sequences underlying SNP markers assigned on the Sharpe et al. (2013) map.

Supplementary Table 5 **Consensus genetic linakge map statistics**. This file contains details of different markers (SSRs and SNPs) and their corresponding positions on different LGs.

Supplementary Table 6 **Phenotypic scores and genotypic data of the lentil germplasm set for the validation of AB resistance markers**. This file contains the phenotypic scores of AB infection on lentil germplasm panel inoculated with *A. lentis* isolate FT12013 alongside genotyped markers for three QTL regions.

## Abbreviations

AB: Ascochyta blight
EST, Expressed sequence tag LG: Linkage group
MAS: Marker-assisted selection
QTL: Quantitative trait locus
RAPD: Random amplified polymorphic DNA
RIL: Recombinant inbred line
SCAR: Sequence characterized amplified region
SNP: Single nucleotide polymorphism
SSR: Simple sequence repeat
R: Resistance.
