# Supplementary material for: SNP-Based Linkage Mapping for Validation of QTLs for Resistance to Ascochyta Blight in Lentil
Source: Front Plant Sci. 2016 Nov 2;7:1604. doi: 10.3389/fpls.2016.01604 (PMC5091049; doi:10.3389/fpls.2016.01604)
Supplement: Supplementary Figure 1 — Comparison between linkage groups of IH × NF, IH × DIG, and NF × DIG maps. This file shows visual representation of the comparison of (A) all LGs in three different maps and the common marker loci between them, (B) LG5 from three maps and the common marker loci between them, (C) LG7 from three maps and the common marker loci between them, (D) LG6 from three maps and the common marker loci between them. White lines represent the corresponding positions of common markers. [file Presentation1.pptx]

## Slide 1
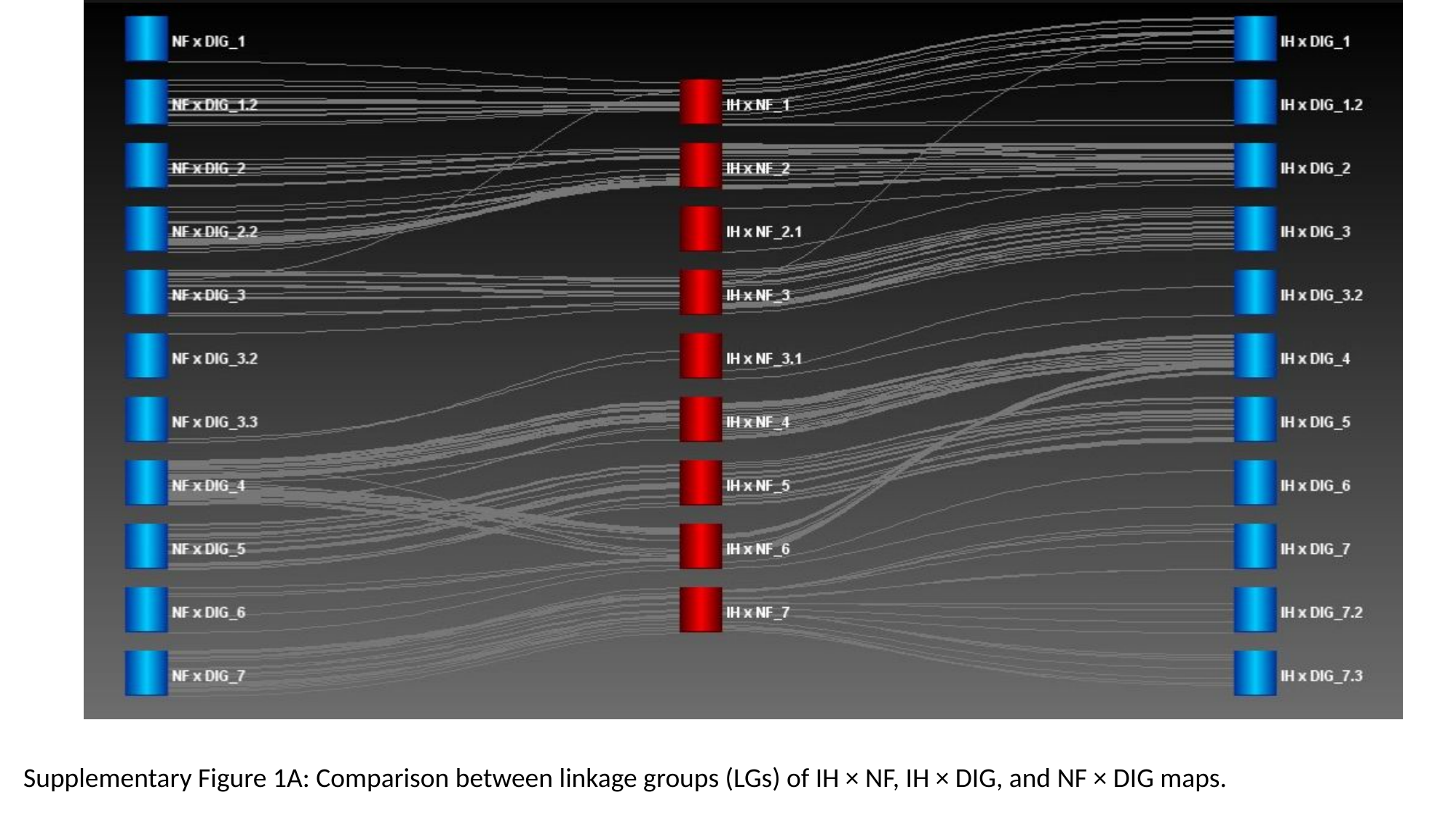

Supplementary Figure 1A: Comparison between linkage groups (LGs) of IH × NF, IH × DIG, and NF × DIG maps.

## Slide 2
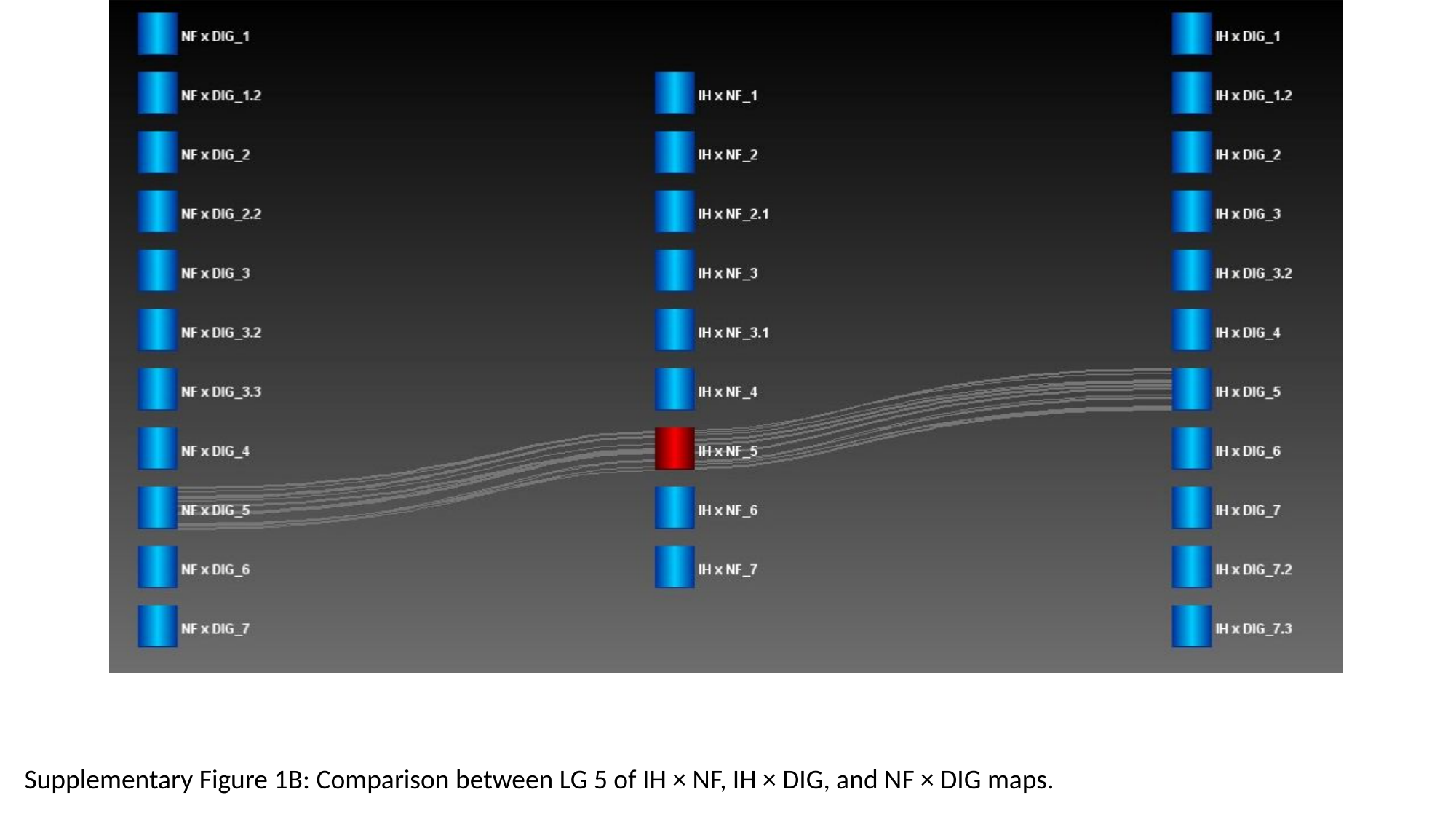

Supplementary Figure 1B: Comparison between LG 5 of IH × NF, IH × DIG, and NF × DIG maps.

## Slide 3
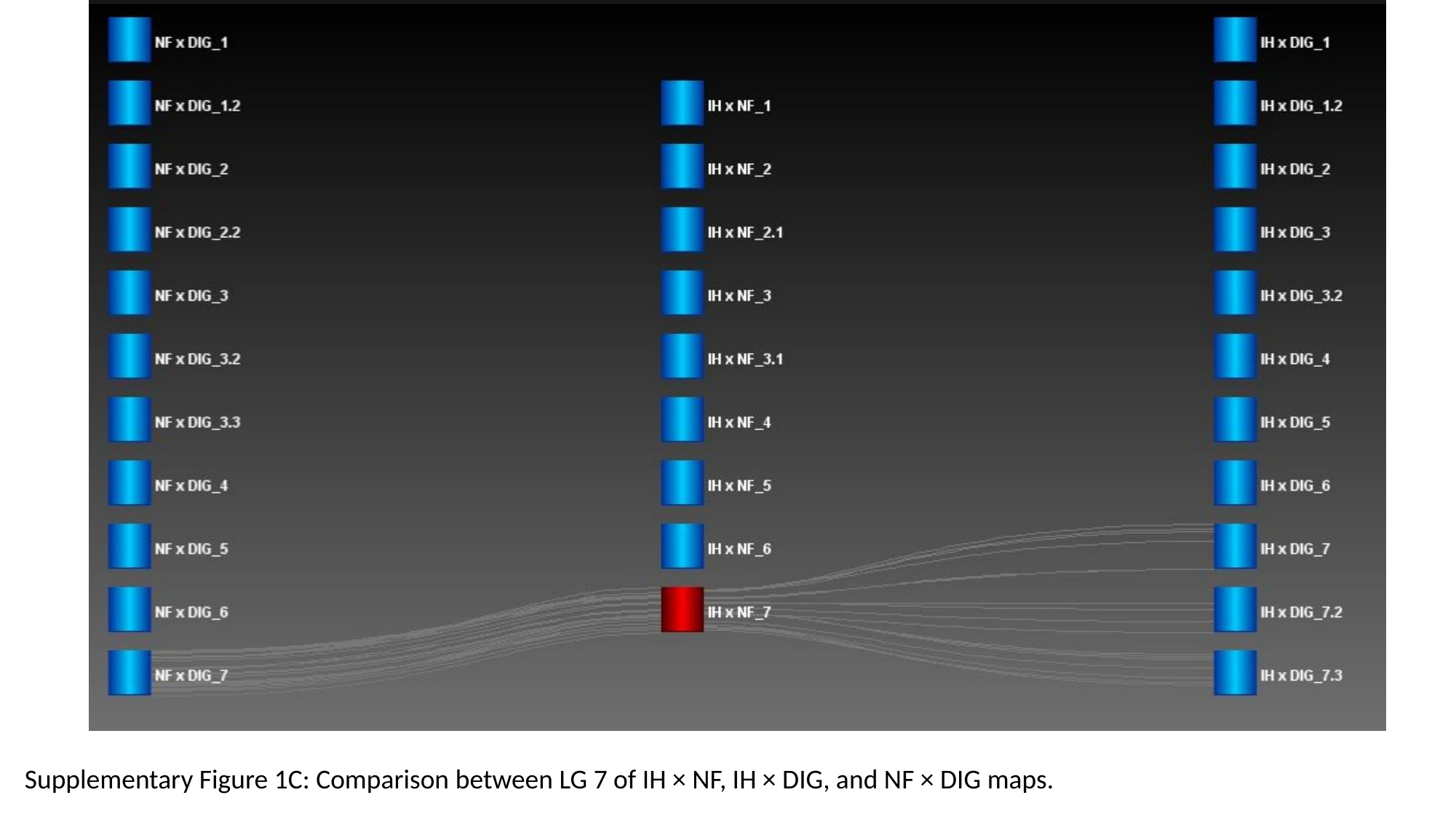

Supplementary Figure 1C: Comparison between LG 7 of IH × NF, IH × DIG, and NF × DIG maps.

## Slide 4
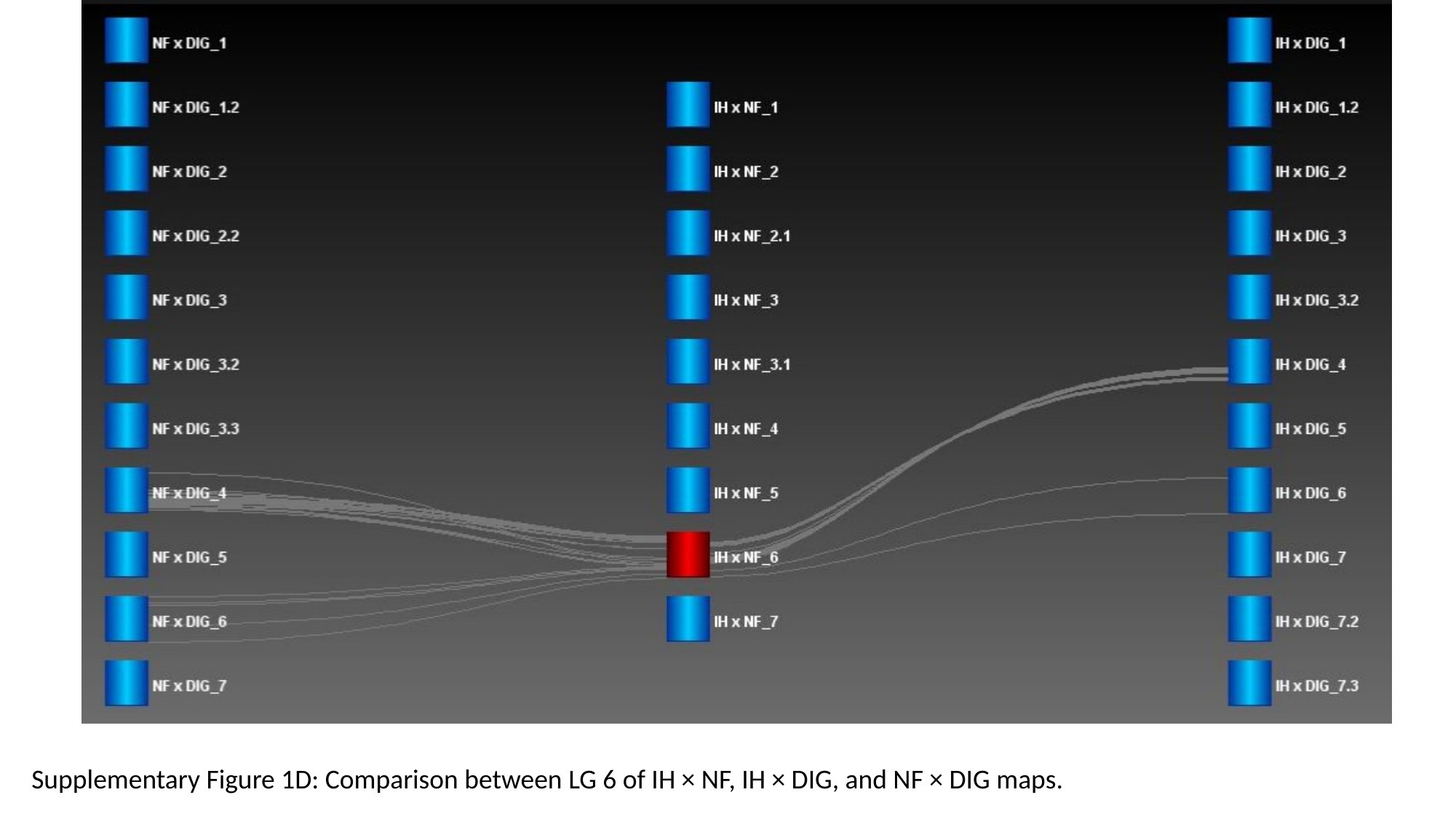

Supplementary Figure 1D: Comparison between LG 6 of IH × NF, IH × DIG, and NF × DIG maps.
