# Supplementary material for: SNP-Based Linkage Mapping for Validation of QTLs for Resistance to Ascochyta Blight in Lentil
Source: Front Plant Sci. 2016 Nov 2;7:1604. doi: 10.3389/fpls.2016.01604 (PMC5091049; doi:10.3389/fpls.2016.01604)
Supplement: Supplementary Figure 2 — Comparison between linkage groups of IH × NF, IH × DIG maps with consensus linkage map. This file shows visual representation of the comparison of (A) all LGs in IH × NF and IH × DIG maps with consensus map and the common marker loci between them, (B) LG5 from three maps and the common marker loci between them, (C) LG6 from three maps and the common marker loci between them. White lines represent the corresponding positions of common markers. [file Presentation2.pptx]

## Slide 1
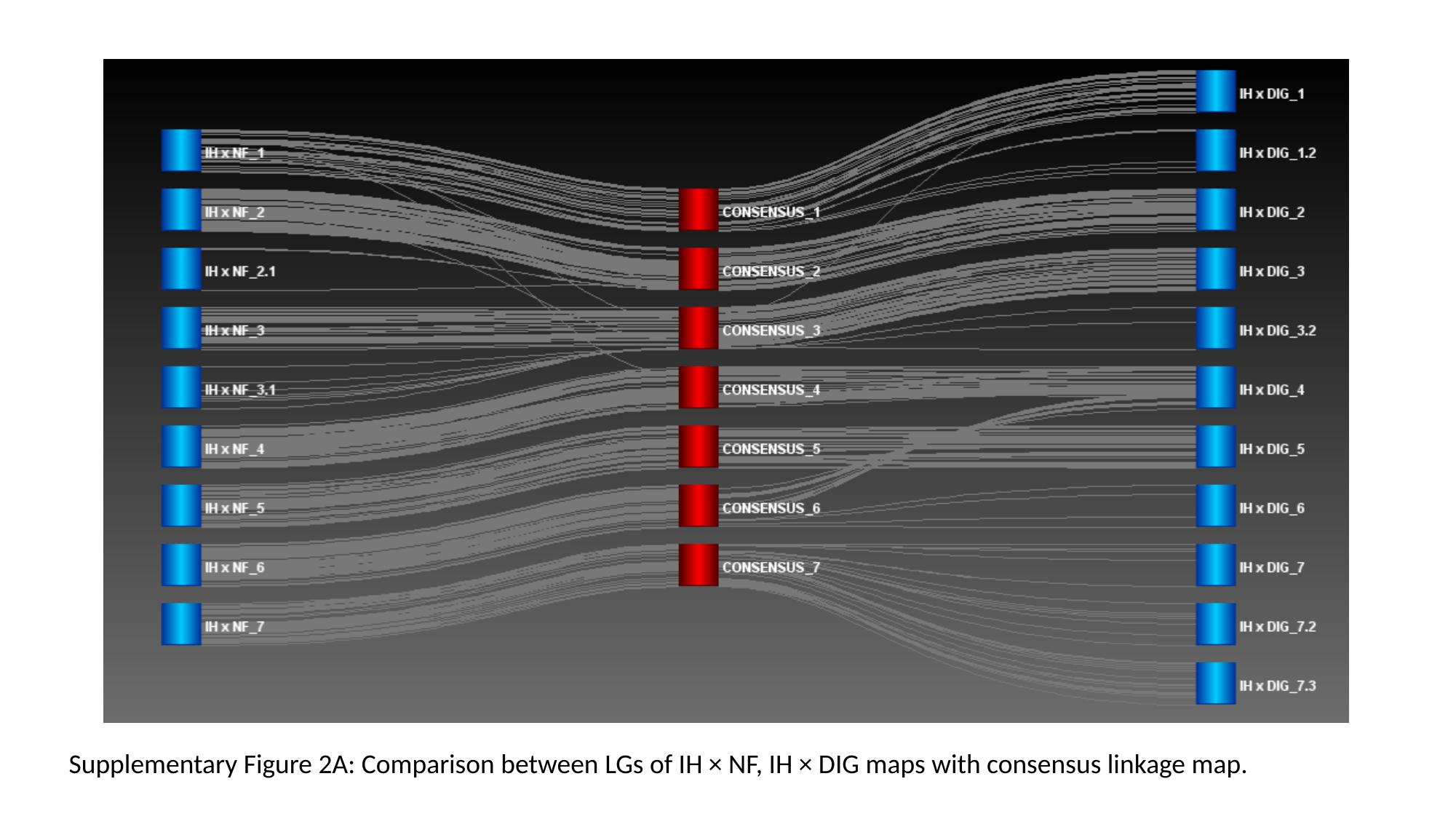

Supplementary Figure 2A: Comparison between LGs of IH × NF, IH × DIG maps with consensus linkage map.

## Slide 2
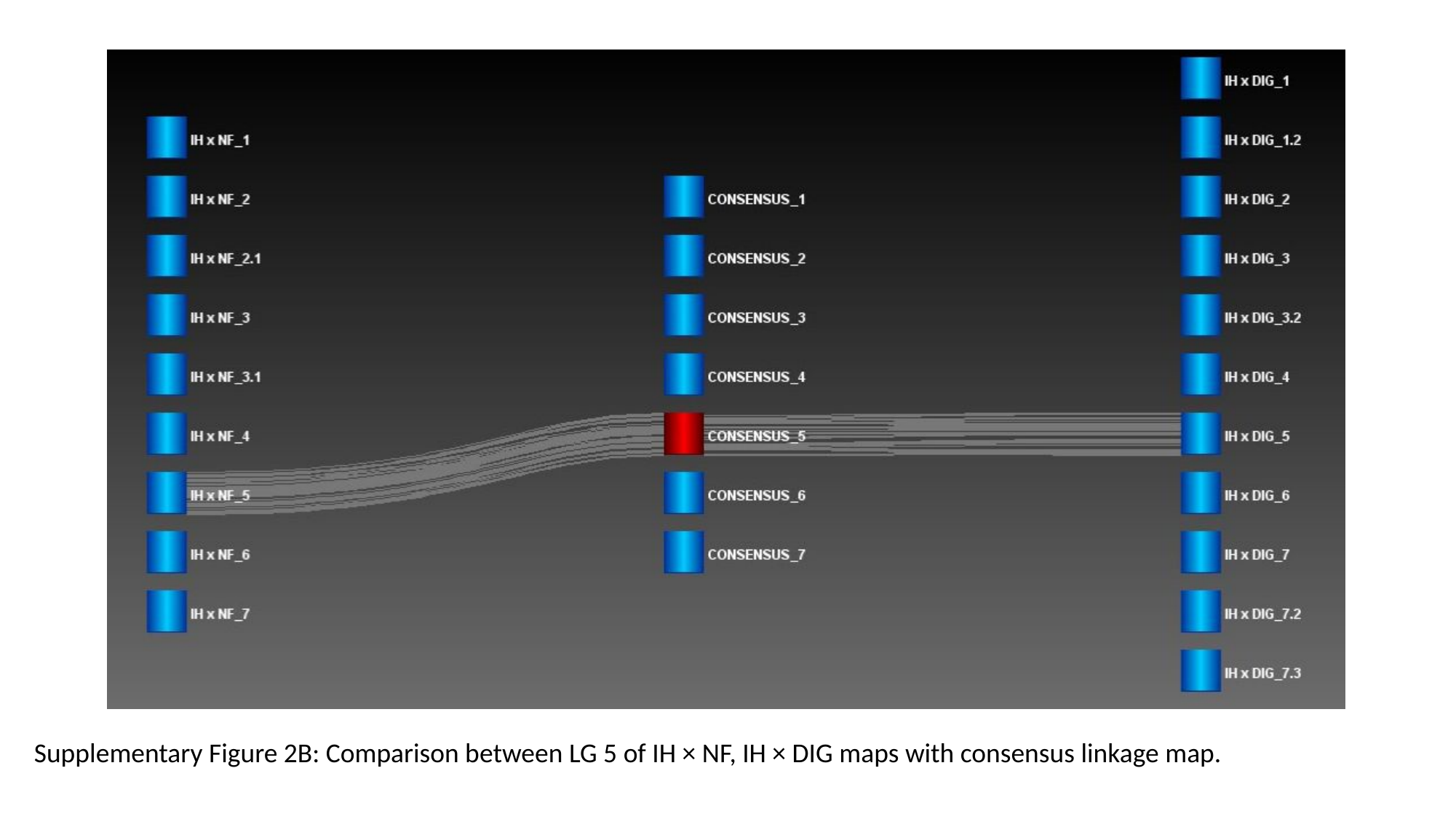

Supplementary Figure 2B: Comparison between LG 5 of IH × NF, IH × DIG maps with consensus linkage map.

## Slide 3
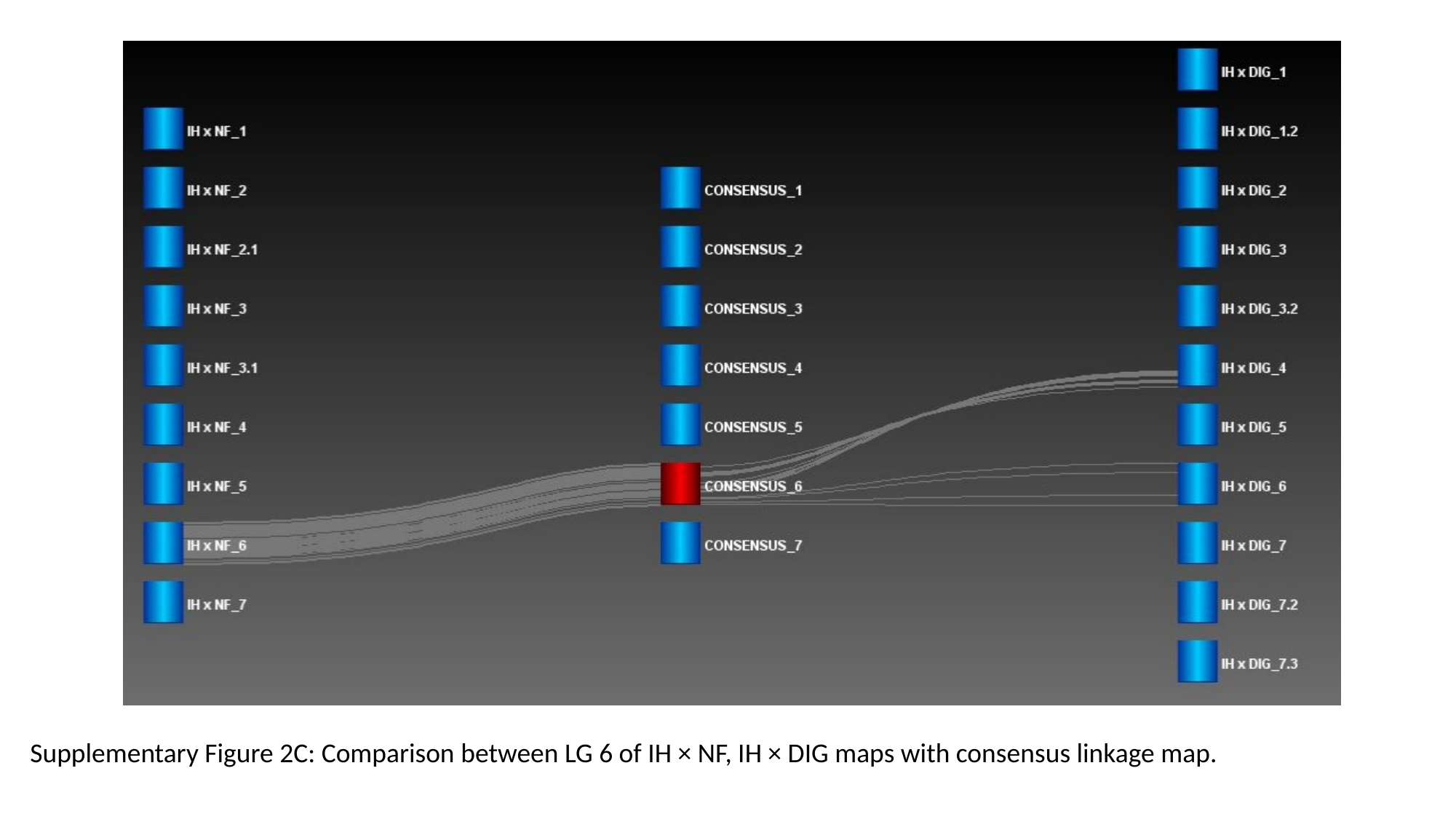

Supplementary Figure 2C: Comparison between LG 6 of IH × NF, IH × DIG maps with consensus linkage map.
