# Supplementary material for: SNP-Based Linkage Mapping for Validation of QTLs for Resistance to Ascochyta Blight in Lentil
Source: Front Plant Sci. 2016 Nov 2;7:1604. doi: 10.3389/fpls.2016.01604 (PMC5091049; doi:10.3389/fpls.2016.01604)
Supplement: Supplementary Figure 3 — Frequency distribution histogram. This file contains frequency histograms generated from AB resistance scores in IH × NF and IH × DIG mapping populations. [file Presentation3.pptx]

## Slide 1
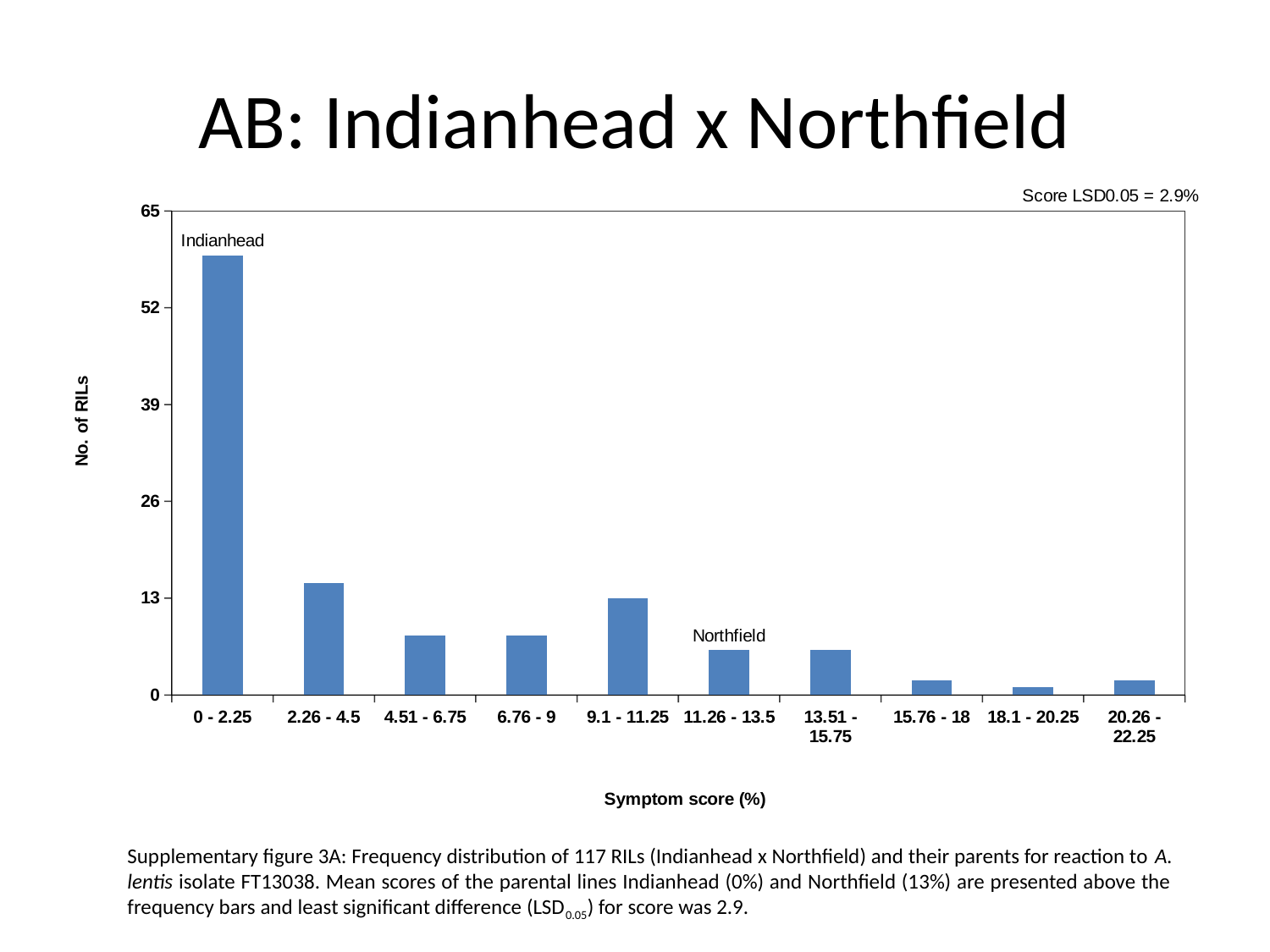

# AB: Indianhead x Northfield
### Chart
| Category | # genotypes |
|---|---|
| 0 - 2.25 | 59.0 |
| 2.26 - 4.5 | 15.0 |
| 4.51 - 6.75 | 8.0 |
| 6.76 - 9 | 8.0 |
| 9.1 - 11.25 | 13.0 |
| 11.26 - 13.5 | 6.0 |
| 13.51 - 15.75 | 6.0 |
| 15.76 - 18 | 2.0 |
| 18.1 - 20.25 | 1.0 |
| 20.26 - 22.25 | 2.0 |Supplementary figure 3A: Frequency distribution of 117 RILs (Indianhead x Northfield) and their parents for reaction to A. lentis isolate FT13038. Mean scores of the parental lines Indianhead (0%) and Northfield (13%) are presented above the frequency bars and least significant difference (LSD0.05) for score was 2.9.

## Slide 2
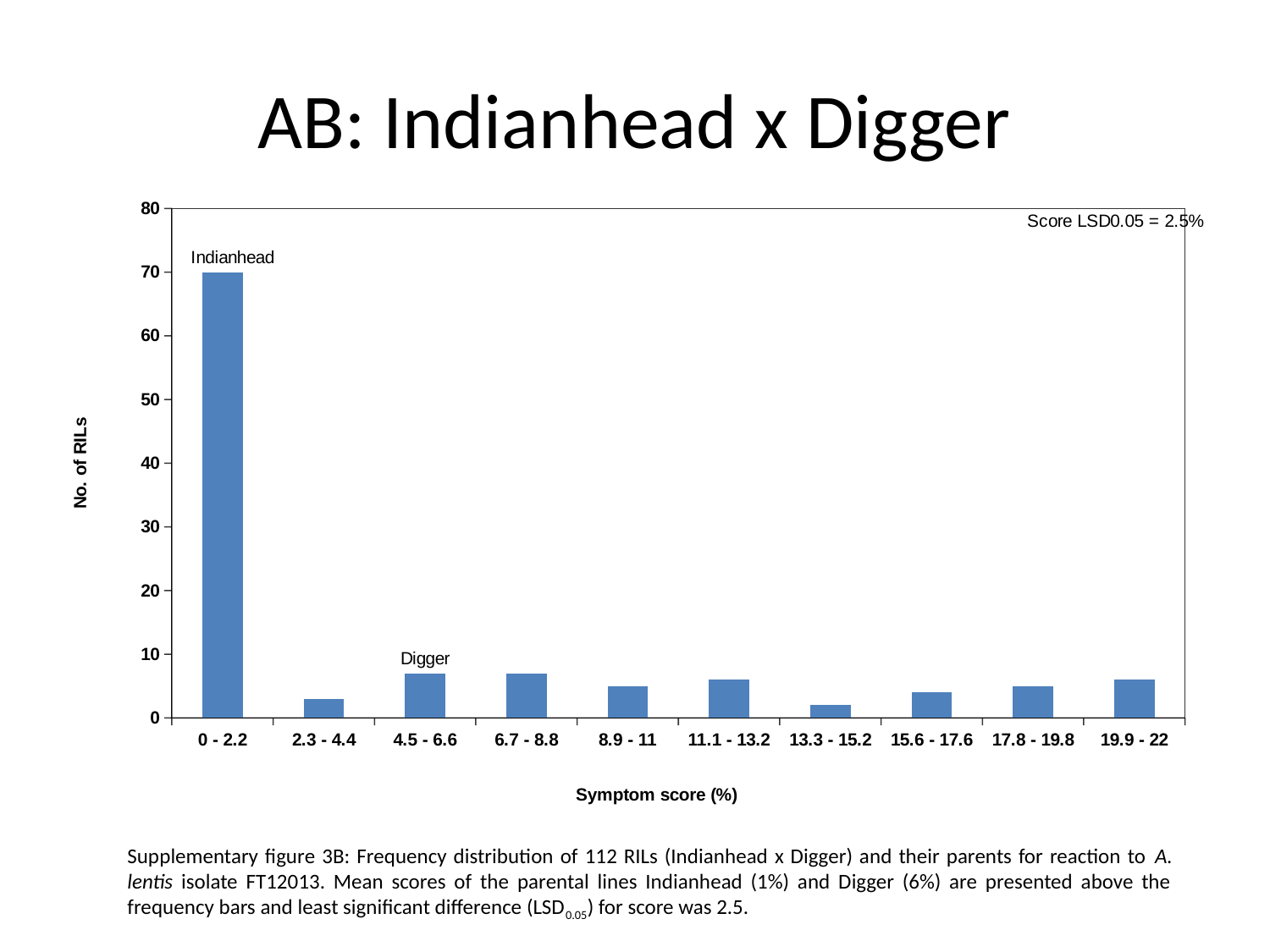

# AB: Indianhead x Digger
### Chart
| Category | No. of RILs |
|---|---|
| 0 - 2.2 | 70.0 |
| 2.3 - 4.4 | 3.0 |
| 4.5 - 6.6 | 7.0 |
| 6.7 - 8.8 | 7.0 |
| 8.9 - 11 | 5.0 |
| 11.1 - 13.2 | 6.0 |
| 13.3 - 15.2 | 2.0 |
| 15.6 - 17.6 | 4.0 |
| 17.8 - 19.8 | 5.0 |
| 19.9 - 22 | 6.0 |Supplementary figure 3B: Frequency distribution of 112 RILs (Indianhead x Digger) and their parents for reaction to A. lentis isolate FT12013. Mean scores of the parental lines Indianhead (1%) and Digger (6%) are presented above the frequency bars and least significant difference (LSD0.05) for score was 2.5.
