# Supplementary material for: SNP-Based Linkage Mapping for Validation of QTLs for Resistance to Ascochyta Blight in Lentil
Source: Front Plant Sci. 2016 Nov 2;7:1604. doi: 10.3389/fpls.2016.01604 (PMC5091049; doi:10.3389/fpls.2016.01604)
Supplement: Supplementary Figure 4 — Frequency distribution histogram. This file contains frequency histograms generated from AB resistance scores from lentil germplasm lines. [file Presentation4.pptx]

## Slide 1
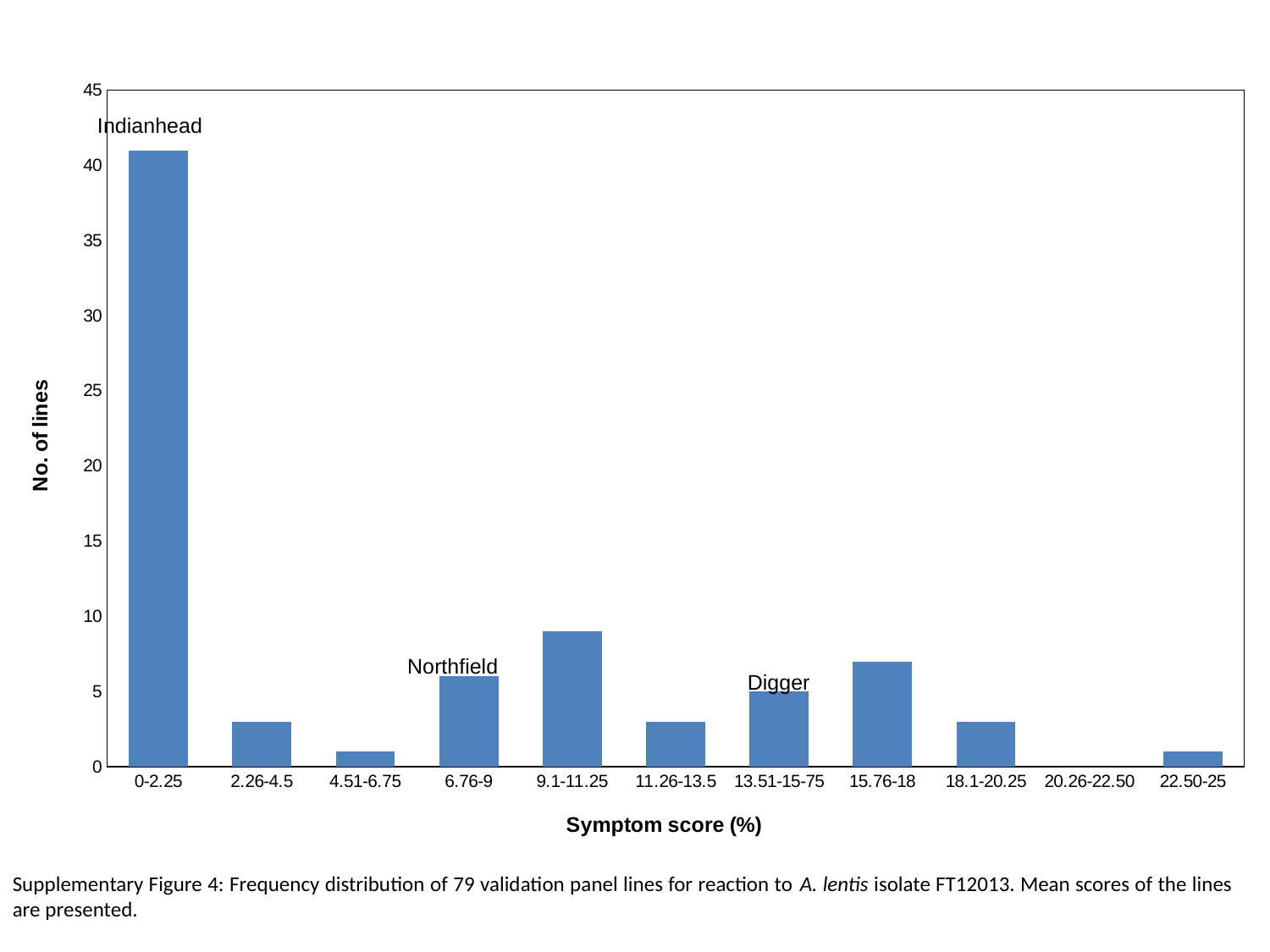

### Chart
| Category | |
|---|---|
| 0-2.25 | 41.0 |
| 2.26-4.5 | 3.0 |
| 4.51-6.75 | 1.0 |
| 6.76-9 | 6.0 |
| 9.1-11.25 | 9.0 |
| 11.26-13.5 | 3.0 |
| 13.51-15-75 | 5.0 |
| 15.76-18 | 7.0 |
| 18.1-20.25 | 3.0 |
| 20.26-22.50 | 0.0 |
| 22.50-25 | 1.0 |Indianhead
Northfield
Digger
Supplementary Figure 4: Frequency distribution of 79 validation panel lines for reaction to A. lentis isolate FT12013. Mean scores of the lines are presented.
